# Uncertainty, epistemics and active inference

**DOI:** 10.1098/rsif.2017.0376

**Published:** 2017-11-22

**Authors:** Thomas Parr, Karl J. Friston

**Affiliations:** Wellcome Trust Centre for Neuroimaging, Institute of Neurology, University College London, 12 Queen Square, London WC1N 3BG, UK

**Keywords:** uncertainty, Bayesian, active inference, neuromodulation, acetylcholine, noradrenaline

## Abstract

Biological systems—like ourselves—are constantly faced with uncertainty. Despite noisy sensory data, and volatile environments, creatures appear to actively maintain their integrity. To account for this remarkable ability to make optimal decisions in the face of a capricious world, we propose a generative model that represents the beliefs an agent might possess about their own uncertainty. By simulating a noisy and volatile environment, we demonstrate how uncertainty influences optimal epistemic (visual) foraging. In our simulations, saccades were deployed less frequently to regions with a lower sensory precision, while a greater volatility led to a shorter inhibition of return. These simulations illustrate a principled explanation for some cardinal aspects of visual foraging—and allow us to propose a correspondence between the representation of uncertainty and ascending neuromodulatory systems, complementing that suggested by Yu & Dayan (Yu & Dayan 2005 *Neuron* **46**, 681–692. (doi:10.1016/j.neuron.2005.04.026)).

## 1. Introduction

In this paper, we address the computational basis for the representation of uncertainty by the brain, and its consequences for epistemic (information gathering^1^) behaviour. We focus on two sources of uncertainty: uncertainty concerning the temporal evolution of environmental states, and uncertainty about the mapping from (hidden) states of the world to sensory observations. Both may arise either from uncertainty inherent in the external world or from noise in neuronal signalling [1]. The first source of uncertainty corresponds to the volatility of state transitions, while the second corresponds to sensory noise and ambiguity. The latter has previously been addressed in the context of predictive coding, in which sensory precision (i.e. inverse variance) modulates the (possibly attentional) gain of ascending prediction errors [2]. This modulatory effect is a direct consequence of (Bayes) optimal evidence accumulation (cf. the Kalman gain of Bayesian filters in engineering). This formulation of attention appeals to the notion of the brain as a statistical organ: an organ that infers the causes of its sensations using internal models of how sensory impressions are generated by continuous states of the world [3,4]. Here, we consider the role of precision in discrete state space models.

Both predictive coding and the (Bayesian) decision processes described in this paper conform to the free energy principle. This principle states that, to prevent its entropy (time average of surprise) growing indefinitely, an agent must maintain an upper bound on surprise [5]. This upper bound is the variational *free energy* of sensory samples [6,7]. The free energy is a function of beliefs about hidden (latent) states, *s*, and a function of observations, *o_τ_*, defined as

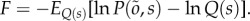


The notation 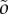 indicates a trajectory of observations through time; i.e. 
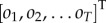
. As written, this equation is very general. To establish its consequences in concrete scenarios, it is necessary to specify a particular form of the generative model, 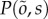, and a factorization of the beliefs, *Q*(*s*), an agent possesses. After the form of the generative model and beliefs have been specified, standard schemes can be used to simulate perception by changing beliefs about hidden or latent states of the world to minimize free energy. This usually involves variational filtering (i.e. Bayesian filtering) for continuous state space models and variational message passing (i.e. belief propagation) for discrete state space models. To simulate action and perception, both beliefs and action minimize free energy. This is known as *active inference*. The novel theoretical contribution of this paper is the inclusion of explicit beliefs about uncertainty in a discrete state space generative model.

We show through simulation that epistemic foraging is heavily influenced by the beliefs an agent has about the volatility and sensory precision of their environment [8]. The temporal dynamics of visual search, including the phenomenon of ‘inhibition of return’, follow naturally from this formulation. The formal contribution of sensory precision to salience dissolves the ‘dark room problem’ [9] associated with active inference, without needing to invoke additional prior beliefs [10]. In the following, we will briefly overview the structure of the generative model we have used previously [11–17]. This model is then supplemented with volatility and precision parameters, to illustrate their role through simulations of visual foraging. These simulations are used to build an intuition behind the phenomenology of Bayes-optimal searches under uncertainty. In the final section, we turn to neuronal implementations of the ensuing ‘precision engineered’ message passing and consider the implications for—and predictions of—empirical studies of attention and neurotransmitter function.

## 2. Markov decision process

**Figure 1.**
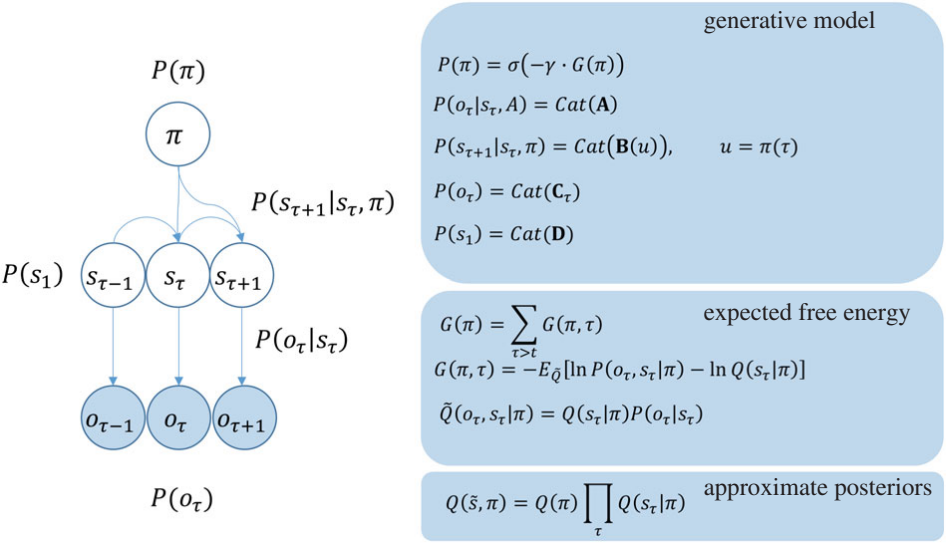
MDP: this Bayesian network *on the left* illustrates the conditional dependencies, and independencies, between the variables in the generative model (see the main text for a description of the variables). The panels *on the right* give the forms of the distributions in the generative model, in addition to defining the expected free energy, and specifying the (mean-field) factorization of the approximate posterior distributions (beliefs) the agent possesses. (Online version in colour.)

A Markov decision process (MDP) is a form of probabilistic generative model [18], defined in a discrete state space. The latent variables of an MDP are hidden states *s_τ_*, and policies, *π*. The conditional dependencies in the model are expressed graphically in figure 1. Hidden states, *s_τ_*, generate observable sensory data, *o_τ_*, with probabilities expressed in a likelihood matrix 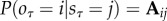. It is this matrix that allows for ‘top-down’ predictions analogous to those in many descriptions of perception [3,4,19,20]. The states evolve through time according to a transition probability matrix, 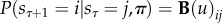, so they depend only on the state at the previous time, and on the policy, *π*, pursued by an agent. It is important to note that, although the first dependency renders the process ‘Markovian’, the dependence on policies of arbitrary lengths breaks this property. In other words, this form of generative model can account for processes with ‘memory’, something that is further enhanced in hierarchical extensions of this model [16,21]. Policies represent sequences of actions, 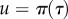, that determine the form of the state transition matrix. To complete the specification of the model for active inference, it is necessary to introduce prior distributions over outcomes, 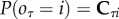, the initial hidden state, 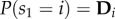, and the policy. Active inference treats policy selection as a Bayesian model selection problem. In other words, policies are selected based upon the free energy, *G*(*π*) expected on pursuing that policy in question [13]. This quantity is defined in figure 1. Using a softmax (normalized exponential) function to convert the expected free energy to a probability distribution, we can write a prior belief over policies as

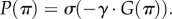

In this equation, *γ* is an inverse temperature parameter, that corresponds to the confidence (or precision) of beliefs about policies. This has been used extensively in previous formulations of active inference, and has been proposed as a computational homologue of dopamine signalling [12,14,15].

## 3. Precision and volatility

To equip the agent with beliefs about the uncertainty in both the transitions of hidden states (i.e. state precision) and the likelihood mapping from hidden states to outcomes (i.e. sensory precision), we now introduce precision parameters to the agent's generative model. These are inverse temperature parameters, analogous to *γ* used for the policy prior. We first augment the likelihood distribution with a sensory precision, *ζ*:

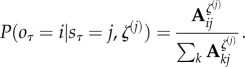


This is a Gibbs measure, commonly expressed as a softmax function [22], for which the denominator is a normalizing constant (partition function). In this equation, *ζ* is the analogue of precision in predictive coding formulations of attentional gain [2]. Note that each value *s_τ_* can take is associated with its own precision. When 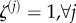, this equation reduces to the formulation in figure 1. The same approach can be followed to define the precision of state transitions (i.e. inverse volatility), *ω*:

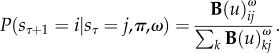


It is simple to extend this, so that *ω*^(*j*)^ is different for each *s_τ_*, as *ζ*^(*j*)^ is, but this is not necessary for the simulations that follow. For simplicity, we will assume 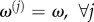 for the remainder of this paper.

**Figure 2.**
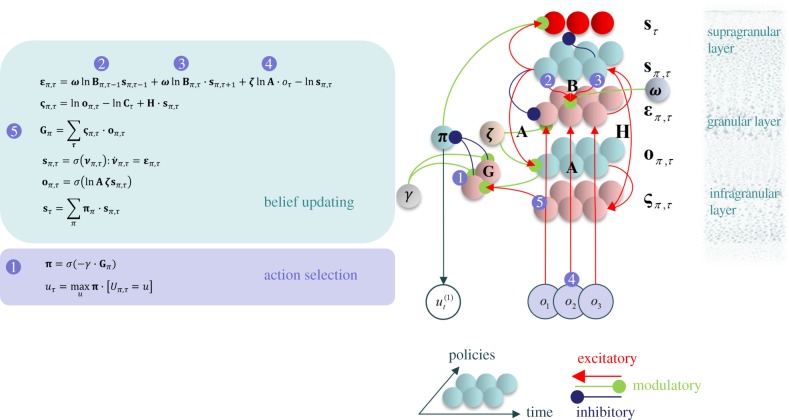
Bayesian belief updates: the panel *on the left* shows the belief updates implied by variational free energy minimization (see appendix A for derivation). *On the right*, these equations are mapped so that the dependencies between beliefs or expectations are shown as connections between neuronal populations. **H** is the entropy of the likelihood matrix. (Online version in colour.)

Given the structure of the MDP shown in figure 1, and the precision parameters defined here, it is possible to express the free energy explicitly, and to find its minimum with respect to each factor in the approximate posterior distribution. In doing so (appendix A), we arrive at the belief update equations in figure 2 for expected states of the world, and expectations about the policies currently being pursued. If these belief update or belief propagation equations are interpreted in terms of message passing in the brain, the resulting connectivity closely resembles that of a cortical column, that participates in a cortico-subcortical loop. Figure 2 illustrates this correspondence by expressing the belief updates for expected states, outcomes and policies 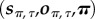, in terms of auxiliary variables; namely, prediction errors and log expectations 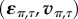 that play the role of neuronal depolarization.

## 4. Simulations

**Figure 3.**
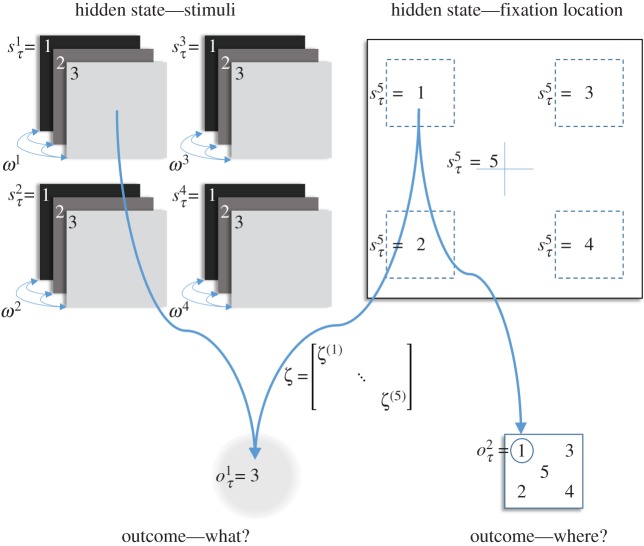
Generative model: the generative model used for the simulations involves five sorts of hidden states. These are four stimuli, and the current eye position. Each stimulus can take one of three identities (illustrated above as dark, medium or light shades). There are five possible fixation locations that correspond to the locations of each stimulus, and the fixation cross. These hidden states map to two outcome modalities: *what* (stimulus identity) and *where* (location). The mapping to the location (i.e. proprioceptive) outcome is an identity mapping from the hidden state representing fixation locations. Each fixation location is associated with a different sensory precision (diagonal elements of *ζ*), mapping to the what (i.e. exteroceptive) outcome. Precisions associated with all other hidden states are 1. Each stimulus hidden state can change identity with a transition precision *ω*. A volatility of *ω*^−1^ = 0 would correspond to an identity transition matrix. Crucially, the prior beliefs about outcomes are uniform, so there are no prior preferences in play. All behaviour resulting from this generative model is therefore purely epistemic and uncertainty reducing in nature. (Online version in colour.)

To illustrate the influence of beliefs about uncertainty on behaviour, the generative model of figure 3 was used to simulate epistemic foraging. The generative model includes four stimuli, whose identity can change stochastically. These stimuli are mapped, noisily, to observable outcomes. Each stimulus is associated with a hidden state that defines its identity. An additional hidden state is the current eye position that determines which of the stimuli is observed. This is associated with an identity mapping to a proprioceptive outcome indicating the current eye position, in a manner consistent with previous MDP models of saccadic eye movements [17]. In brief, this means that given the hidden states (namely, where the agent looks and the states of the stimulus at that position), one can generate probabilistic outcomes (proprioceptive information about where the agent is looking and exteroceptive outcomes reporting stimulus identity).

The behaviour observed in the simulations can be explained by referring to the agent's beliefs about policies. As shown in figure 1, a system that engages in active inference selects policies with a low expected free energy. A rearrangement of the equation for expected free energy at a future time gives the following

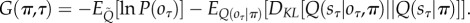


**Figure 4.**
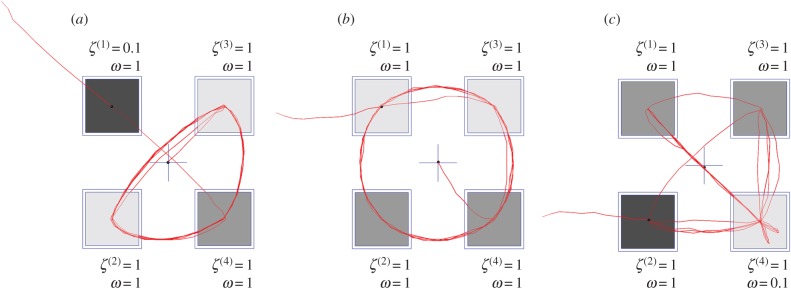
Simulated visual foraging. These plots show the simulated eye tracking data (19 saccades) for the MDP, and the influence of varying sensory precision and volatility. (*a*) The situation in which precision is (believed to be) low when the top left location is foveated is shown. (*b*) The precisions for each location are equal, as are the volatilities for each stimulus. (*c*) The lower right stimulus is associated with increased volatility. (Online version in colour.)

The first term here corresponds to prior preferences. These are uniform across all outcomes in these simulations. The second term corresponds to epistemic value or salience, and it is this that drives the active sampling of a visual scene [23,24]. The greater the change expected in beliefs with and without future outcomes, the lower the expected free energy. In other words, the salience reflects the expected information gain or resolution of uncertainty about states of the world. For a location associated with a low sensory precision, an observation is unlikely to elicit a substantial change in the posterior, so a saccade to such a location is less likely to be selected. This behaviour is illustrated in figure 4*a*, which shows a simulated sequence of actions (i.e. saccadic eye movements) over eight sensory samples.

**Figure 5.**
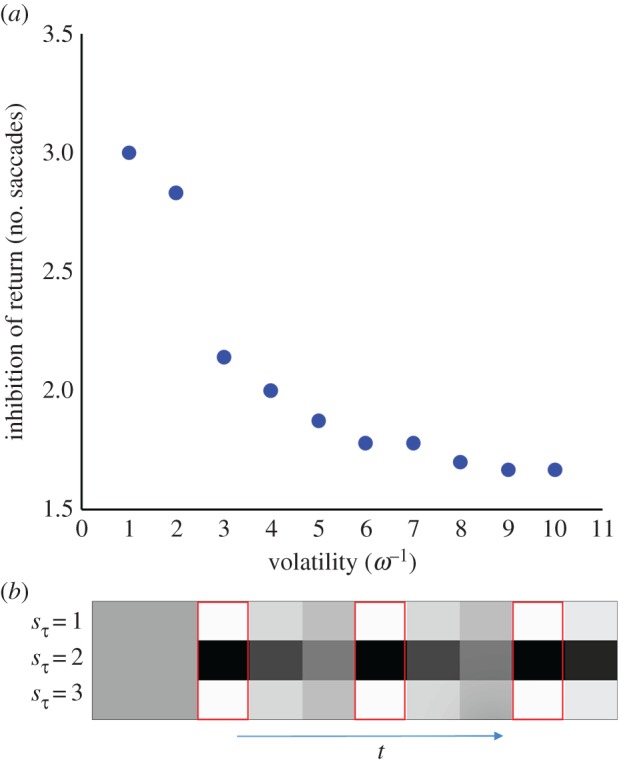
Simulated neuronal encoding and inhibition of return. (*a*) The influence of volatility beliefs on the simulated inhibition of return (quantified by the average number of saccades between fixations) for one of the stimulus locations. This is consistent with empirical data using noradrenergic pharmacological manipulations [26], and pupillometry [27,28]. (*b*) The simulated neuronal encoding over time for three units, each representing a different hypothesis about the identity (1, 2 or 3) of one of the hidden states. Darker shades indicate greater firing rates. The red boxes indicate the times during which the location corresponding to this hidden state is foveated. (Online version in colour.)

Heuristically, this is why a well-lit room, with precise sensory information, is preferable to a dark room; i.e. precise sensory cues that resolve ambiguity have greater epistemic affordance and are more likely to be sampled. More colloquially, this sort of behaviour recapitulates the joke about the drunkard looking for a lost key under a streetlamp (the ‘Streetlight effect’) [25]. Notably, the drunkards ‘cognitive bias’ is entirely Bayes optimal on an active inference view. The greater frequency of saccades to stimuli with a higher volatility (figure 4*b*) can be similarly explained. On making an observation concerning a stimulus, the expected posterior at the next time step should be very close to the current belief, for that stimulus. Recent observations are thus associated with a lower salience that then gradually increases over time, as the probability that the hidden state has transitioned to a new value increases (figure 5). In other words, knowing the state of a stimulus in a particular location means there is no further information to be gained by sampling that location and it loses its salience. Note that salience is an attribute of both the world and the agent's beliefs about the world. However, if the stimulus can change, the salience of its location will increase slowly over time with uncertainty about its current status.

This phenomenon is consistent with the ‘forgetting slopes’ determined by calculating the error in reports about a stimulus at different times following presentation [29], and with theoretical analyses of the properties of the attractor networks used to explain the maintenance of working memory signals [30]. The concept of ‘inhibition of return’ [31,32] naturally emerges from this formulation, as an agent becomes less likely to return to the same location for a temporally limited period following a fixation. A stimulus with high volatility (figure 4*b*) would then have a shorter inhibition of return, leading to a greater frequency of fixation. Formulating volatility in this way means that the *ω* parameter can be estimated from real subjects simply by measuring the length of the inhibition of return.

In summary, using a very simple but plausible formulation of active inference in the context of searching a simple visual scene, we find a natural explanation for two key phenomena in visual search; namely the attractiveness of salient, uncertainty reducing target locations and inhibition of return that depends upon the volatility of a visual scene. Crucially, both of these phenomena rest on encoding the uncertainty or precision of state transitions and the generation of (visual) outcomes from hidden states. In what follows, we now consider the neuronal encoding of precision and the intimate relationship between salience, attention and epistemic affordance.

## 5. The neurobiology of precision

We have presented some elementary simulations to illustrate fairly straightforward phenomena that emerge under active (Bayesian) inference; namely, that the encoding of uncertainty or precision nuances perception and action in a fundamental but sensible way. In what follows, we revisit the encoding of uncertainty from the point of view of plausible neuromodulatory transmitter systems, the implicit computational anatomy and implications for neuropsychology. This treatment is not a review of the psychology of prediction under uncertainty but a selective survey of empirical findings that speak to the computational architecture of the preceding sections.

### 5.1. The pharmacology of uncertainty

While we have contrasted sensory uncertainty with volatility, other authors have emphasized the difference between ‘expected uncertainty’ and ‘unexpected uncertainty’ [33]. These two dimensions of classification align more closely than might initially appear. ‘Expected uncertainty’ is framed in terms of a belief that a cue has low validity. In other words, the mapping from a task context (hidden states) to an informative observation is believed to be imprecise. This exactly corresponds to imprecision *ζ*^−1^. ‘Unexpected uncertainty’ is described in terms of stochastic changes to the task context. Such changes are necessarily determined by a probability transition matrix, so this form of uncertainty is associated with *ω*^−1^. Just as *γ* has been associated with dopaminergic signalling [15], these two forms of uncertainty correspond to the activity in neuromodulatory systems; specifically, the cholinergic [34,35] and noradrenergic systems [36].

Nicotinic acetylcholine receptors are expressed presynaptically at thalamocortical synapses in cortical layers 3 and 4 [37,38], the laminar targets of first-order thalamic nuclei [39]. These have been shown to modulate sensory gain [40,41] in the visual system. This is consistent with *ζ* in both the anatomy shown in figure 2, in which it modulates the synapses carrying sensory data to the cortex, and in the functional role implied by the update equations. Acetylcholine receptors are also found in other cortical layers [42,43], including deeper layers, which is again consistent with figure 2. Muscarinic receptors also appear to have an important role in attentional gain mechanisms [44].

Noradrenaline [33,36] has been proposed to signal uncertainty about state transitions. It has also been implicated in modulation of the balance between exploitative and exploratory (epistemic) behaviours [45]. Our simulations demonstrate the consistency of these propositions, as a greater transition uncertainty means that the salience or epistemic affordance of a particular sampling of the environment increases at a faster rate in a volatile context. Pupillary data, associated with catecholamine signalling [46], provide additional support to the hypothesis that noradrenaline is involved in signalling *ω*, as dilatation occurs during the delay period of working memory tasks [47]. Such tasks require the maintenance of beliefs about a given stimulus identity, that correspond to the belief that the stimulus identity will not change throughout the delay period (i.e. that the precision of state transitions *ω* is high). The relationship between pupillary diameter and central noradrenaline offers a means to test the predictions of this neurochemical model of precision signalling. By manipulating the volatility of stimulus transitions, we can test the hypothesis that increases in *ω* are associated with pupillary constrictions, and that increases in *ω* are accompanied by dilatations. Crucially, we would not expect modulations in other precision parameters to induce these changes.

### 5.2. The neuroanatomy of uncertainty

**Figure 6.**
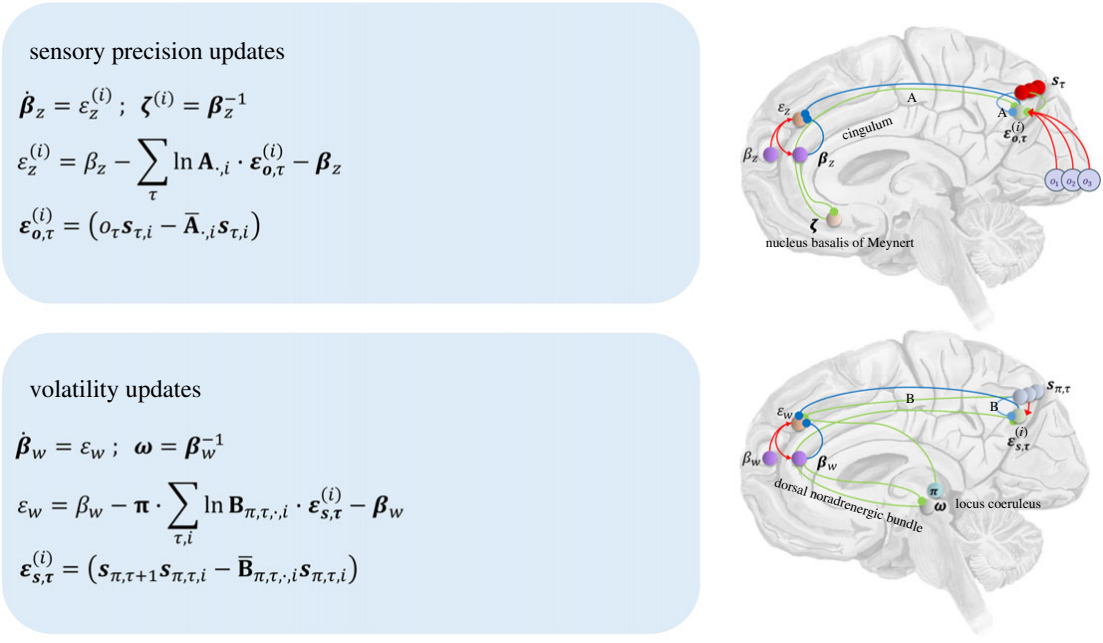
Uncertainty updates and neuroanatomy. *On the left,* the Bayesian update equations for the representations of sensory precision and volatility are shown. These are derived in appendix A, and are presented here as gradient ascents. *On the right*, the variables in these equations are assigned to neuronal populations. This assignment is speculative, but is intended to show how the updates could be implemented in the brain. It is also consistent with the observations in the main text that ascending neuromodulatory systems are likely to signal changes in precision or volatility to the cortex, and that prefrontal regions are well placed to compute the necessary signals. (Online version in colour.)

Having parametrized both sensory precision and volatility, we are now in a position to derive Bayes optimal updates for these parameters (see appendix A). This means that the agent can infer the precision of its environment, in terms of both likelihood mappings and state transitions. The resulting update equations are shown in figure 6. This figure additionally shows how the Bayesian updates for *ζ* and *ω* could map onto the connectivity between the cortex and the noradrenergic and cholinergic systems. These are related to cortical areas via the cingulum, and the dorsal noradrenergic bundle. Damage to the latter has been linked to deficits in epistemic behaviour [48,49] and attentional set-shifting [50]. Disruption of the dorsal noradrenergic bundle has also been associated with impaired extinction of a conditioned stimulus [51], perhaps reflecting a representation of very low volatility.

If volatility is signalled by noradrenaline, the networks computing this quantity should interact with the locus coeruleus, a noradrenergic brainstem nucleus that projects too much of the cortex [52]. Anterograde tracing has demonstrated that the prefrontal cortex is a source of projections to the locus coeruleus [53]. Pharmacological manipulations [54] show that these projections influence the activity of brainstem noradrenergic neurons. Specifically, inactivation of frontal regions causes a sustained increase in locus coeruleus firing. This makes these regions good candidate sites for the computation of volatility. In figure 6, volatility is determined using volatility prediction errors in the prefrontal cortex, making use of projections from sensory areas (that carry state prediction errors). The locus coeruleus then sends a precision (inverse volatility) signal to the cortex. Given the close association between central noradrenaline and pupillary diameter [46], the dynamics of the Bayesian updates given here could be incorporated into an MDP-based generative model of pupillary data, first to establish the validity of the updates as a description of noradrenergic signalling, and then as part of an generative model of empirical responses that can be elicited experimentally [55].

Prefrontal regions also project to the basal forebrain [56], the primary source of cholinergic projections to the cortex. To reach the cortex, fibres from the basal forebrain pass the corpus callosum rostrally, before joining the cingulum [43]. Cholinergic axons leave this white matter bundle to diffusely innervate the cerebrum. Figure 6 shows that sensory precision can be calculated in a manner analogous to volatility. Outcome prediction errors in sensory areas are propagated to frontal regions that calculate a precision-related prediction error. This is used to generate a signal to the nucleus basalis of Meynert; a forebrain nucleus that provides a cholinergic signal to sensory cortices.

### 5.3. The neuropsychology of uncertainty

The disruption of the dopaminergic modulation of policy precision can result in disease states, including Parkinson's disease [15,57,58], and schizophrenia [59–61]. Similarly, the neurotransmitter systems associated here with sensory precision and volatility are disrupted in a range of neuropsychiatric disorders. Depletion of acetylcholine is associated with Alzheimer's disease [62,63], while disruptions of noradrenaline signalling are thought to contribute to anxiety [64], post-traumatic stress disorder [65], depression [66] and Wernicke–Korsakoff encephalopathy [67,68]. Additionally, the lateral asymmetry of noradrenergic projections in the forebrain [69], reflected in pupillary responses [70], hints at a role in hemineglect [71]. A formal description of the computational processes that are disrupted in these disorders allows for the development of a computational phenotyping [55] of patients. This may aid in the characterization of defective neurophysiology, making use of the process theory [12] associated with active inference.

## 6. Discussion

In order to act optimally in an ambiguous and volatile world, it is necessary to possess a generative model that incorporates key forms of uncertainty. Through their influence on salience—a component of the expected free energy of a policy—uncertainty can have a profound influence on epistemic behaviour. For example, volatile contingencies call for a shorter inhibition of return, while imprecise sensory information is inherently less epistemically valuable. The parametrization in this paper facilitates the derivation of Bayesian updates for precision parameters—that could be implemented by a network of sensory, prefrontal and subcortical structures. Functionally and anatomically, the ascending cholinergic and noradrenergic systems are plausible neurobiological substrates for the computational processes described in this paper.

The story on offer here provides a coherent and formal account of neuromodulation in the brain that is broadly consistent with previous neurobiological accounts of perception and decision-making [8,72,73]. In brief, there are three fundamental sorts of beliefs that determine behaviour: (i) beliefs about outcomes given hidden or latent states of the world, (ii) beliefs about states of the world, and (iii) beliefs about policies given states of the world. Each of these sets of beliefs is equipped with an uncertainty or precision that may be encoded by specific modulatory neurotransmitter systems. The evidence reviewed above—and in [8,33]—speaks to the following (summarized in table 1): (i) cholinergic systems encode the precision of beliefs about outcomes given states of the world (cf. attention and expected uncertainty); (ii) noradrenergic systems encode the precision of state transitions (cf. volatility and unexpected uncertainty); and (iii) dopaminergic systems encode the precision of beliefs about policies (cf. action selection). The coherent aspect of this account rests on the fact that all three systems play the same computational role; namely, an encoding of precision. Furthermore, all three neurotransmitter systems have the same basic effects on synaptic transmission; namely, a neuromodulatory gain control.

**Table 1.** Summary of the functional anatomy of precision.

precision	functional role	neurotransmitter system	neuroanatomy
*ζ*	encoding the precision of outcomes given hidden states (cf. attention and expected uncertainty [34,35])	cholinergic	nucleus basalis of Meynert
*ω*	encoding the precision of state transitions (cf. volatility and unexpected uncertainty [36])	noradrenergic	locus coeruleus
*γ*	Encoding the precision of beliefs about policies (cf. action selection [15])	dopaminergic	substantia nigra pars compacta, ventral tegmental area

## Endnote

1 As opposed to epistemic in the philosophical sense.

## Data accessibility

The simulations in this paper have been performed using the standard software routine spm_MDP_VB_X.m. This is freely available as Matlab code as part of academic software which can be downloaded at http://www.fil.ion.ucl.ac.uk/spm/.

## Appendix A

**A.1. Belief update equations**

The variational free energy for the MDP model described in the main text is

Here, the ∼ notation denotes sequences of outcomes and hidden states over time. Treating policy selection as a process of Bayesian model selection, we can consider the free energy of each policy

Using the mean-field approximation , and factorizing the joint distribution using the conditional independencies in the generative model, this can be written as:

Taking the variational derivative of this with respect to beliefs about hidden states at a particular time we have,

Setting the variational derivative to zero, the variational solution is

Where *Z* is a partition function (i.e. a constant). In terms of the model parameters and expectations

To express this as a gradient ascent, we define an error term as the difference between the current belief and the variational solution, and change the current belief to minimize this error:

To determine the form of ***ɛ****_π,τ_*, we have specified the form of the distributions contributing to the variational solution. In the above, the log transition probability takes the form , while the log likelihood is , where

There are partition functions associated with these distributions; however, these are constant with respect to the belief about the current state. This means they can be omitted in the derivation of the Bayesian belief updates for hidden states. Substituting the log probability distributions into the above, the error can be written,

This is the form used for belief updating in figure 1.

**A.2. Derivation of Bayesian updates for precision parameters**

In deriving belief updates for precision parameters, the partition functions cannot be omitted, as they are functions of the parameters being updated. We assume that the precision parameters are distributed according to gamma distributions, and follow a similar line of reasoning to that used to derive updates for policy precisions in previous papers. The prior distribution over the precision parameters is then:

The approximate posterior distributions have the same (gamma distribution) form and we will use a bold *β* hyperparameter to distinguish between the sufficient statistics of the posterior and prior above. A useful property of the gamma distribution, when parametrized in this way, is the following

Having defined these distributions, we can write the variational free energy

Which can be expressed in terms of sufficient statistics (omitting constants),

where

correspond to normalized probability matrices. Taking the partial derivative with respect to the expected precision of state transitions gives:

Solving for zero gives the volatility expectations:

The updates for the sensory precision are obtained in the same way:

Expressing these updates as biologically plausible gradient ascents, the resulting equations are

where the errors are

The partial derivatives here can be expressed explicitly in terms of weighted prediction errors

These are equivalent to the forms in figure 6, which are expressed to emphasize the plausibility of their neuronal implementation.
